# The Evidence for the Use of Osteobiologics in Hybrid Constructs (Anterior Cervical Discectomy and Fusion and Total Disc Replacement) in Multilevel Cervical Degenerative Disc Disease: A Systematic Review

**DOI:** 10.1177/21925682221150795

**Published:** 2024-02-29

**Authors:** Thomay-Claire A. Hoelen, Paul C. Willems, Arjan Loenen, Hans Jörg Meisel, Jeffrey C. Wang, Amit Jain, Zorica Buser, Jacobus J. Arts, AO Spine Knowledge Forum Degenerative

**Affiliations:** 1 Laboratory for Experimental Orthopaedics, Department of Orthopaedic Surgery, CAPHRI, 199236 Maastricht University Medical Center, Maastricht, the Netherlands; 2 Orthopaedic Biomechanics, Department of Biomedical Engineering, 425625 Eindhoven University of Technology, Eindhoven, the Netherlands; 3 Department of Neurosurgery, BG Klinikum Bergmannstrost, Halle, Germany; 4 Department of Orthopaedic Surgery, Keck School of Medicine, University of Southern California, Los Angeles, CA, USA; 5 Department of Neurosurgery, Keck School of Medicine, University of Southern California, Los Angeles, CA, USA; 6 Department of Orthopaedic Surgery, 1500 Johns Hopkins University, Baltimore, MD, USA; 7 Department of Orthopedic Surgery, NYU Grossman School of Medicine, New York, NY, USA; 8 Gerling Institute, Brooklyn, NY, USA

**Keywords:** cervical degenerative disc disease, hybrid surgery, intervertebral disc degeneration, osteobiologics, range of motion, articular, total disc replacement

## Abstract

**Study Design:**

Systematic review

**Objective:**

Examine the clinical evidence for the use of osteobiologics in hybrid surgery (combined anterior cervical discectomy and fusion (ACDF) and total disc replacement (TDR)) in patients with multilevel cervical degenerative disc disease (DDD).

**Methods:**

PubMed and Embase were searched between January 2000 and August 2020. Clinical studies investigating 18-80 year old patients with multilevel cervical DDD who underwent hybrid surgery with or without the use of osteobiologics were considered eligible. Two reviewers independently screened and assessed the identified articles. The methodological index for non-randomized studies (MINORS) tool and the risk of bias (RoB 2.0) assessment tool were used to assess risk of bias. The Grading of Recommendations, Assessment, Development and Evaluations (GRADE) was used to evaluate quality of evidence across studies per outcome.

**Results:**

Eleven studies were included. A decrease in cervical range of motion was observed in most studies for both the hybrid surgery and the control groups consisting of stand-alone ACDF or TDR. Fusion rates of 70-100% were reported in both the hybrid surgery and control groups consisting of stand-alone ACDF. The hybrid surgery group performed better or comparable to the control group in terms of adjacent segment degeneration. Studies reported an improvement in visual analogue scale for pain and neck disability index values after surgery compared to preoperative scores for both treatment groups. The included studies had moderate methodological quality.

**Conclusions:**

There is insufficient evidence for assessing the use of osteobiologics in multilevel hybrid surgery and additional high quality and controlled research is deemed essential.

## Introduction

Cervical degenerative disc disease (DDD) is commonly seen in clinical practice and is known to progress over time. Cervical DDD can cause pain and/or neurological deficits and thus seriously impact quality of life.^1^ Anterior cervical discectomy and fusion (ACDF) is a well-established and effective surgical technique for treating cervical DDD.^2-4^ However, in case of multilevel cervical DDD, it has been suggested that multilevel fusion could be disadvantageous due to the range of motion constraints and the potential to induce adjacent segment degeneration (ASD) in an accelerated manner.^4^

Total disc replacement (TDR), also known as cervical disc arthroplasty (CDA), has commonly been used as an alternative to ACDF. TDR aims to maintain cervical range of motion (ROM) of the affected segments and consequently reduces ASD.^5^ In case of multilevel cervical DDD, however, TDR is related to a higher risk of disc prosthesis complications and increased medical costs compared to ACDF. As a result, performing multilevel TDR can be less feasible in clinical practice.^4-7^

With the purpose to overcome these challenges in multilevel cervical DDD, hybrid surgery (combining ACDF and TDR), has been introduced. Hybrid techniques aim to combine the most effective elements of both surgical interventions. Thereby, the degenerative status of each cervical level can be considered individually with the aim of maintaining sufficient cervical ROM as well as sufficient stability of the cervical spine.^4,8^ When stabilizing a cervical level, it is essential to attain a decent fusion, as high rates of radiological fusion correlate with improved postoperative outcomes.^9^ The rate of fusion can be stimulated by the use of bone grafts or osteobiologics. Although autologous bone grafts are considered the ‘gold standard’, there are inherent disadvantages such as complications that may occur at the donor site (pain, hematoma, fracture), prolonged operation time, and limitations in availability.^10,11^ Allografts may tackle these disadvantages but are associated with a higher risk of infections or immunogenic rejection as well as lower donor graft quality. Moreover, allografts do not contain osteogenic properties.^10^

There is growing evidence for the use of osteobiologic graft substitutes to surmount these limitations. Osteobiologic graft substitutes are a group of synthetic biomaterials that mimic the desired traits of osteoinductivity, osteoconductivity and osteogeneity in varying degrees. Thus, they are often applied to promote spinal fusion as well as to provide structural support.^10,12^ Common osteobiologic graft substitutes include ceramics, polymers, bioactive peptides, bone morphogenetic proteins (BMPs) and demineralized bone matrix (DBM). The use of osteobiologics, as bone graft extender and as stand-alone bone graft substitute is rapidly expanding.^10,11,13^ The properties of osteobiologics determine its effectiveness as bone graft substitute. The type of bone graft may be essential for the extent of bone growth at the fused level and consequently may impact the overall effectiveness of hybrid surgery. Therefore, the objective of this systematic review was to determine the evidence for the use of osteobiologics in hybrid surgery in patients with multilevel cervical DDD.

## Methods

### Protocol Registration

This study was registered in the International prospective register of systematic reviews (PROSPERO) under the registration number: CRD42020209665. This manuscript has been written according to the Preferred Reporting Items for Systematic Review and Meta-analysis (PRISMA) statement.^14^ A PRISMA checklist is provided in Supplementary Appendix I.

### Eligibility Criteria

Studies investigating patients aged between 18 and 80 years, suffering from degenerative cervical discs who had undergone multilevel (2-4 levels) hybrid surgery with or without osteobiologics were considered eligible. Peer-reviewed articles of randomized or non-randomized controlled trials, cohort studies, and prospective case series (n > 20) were included. Articles published before the year 2000 were excluded. The main outcome of interest was radiographical assessment of the cervical ROM. Studies including participants with a history of tumours, infection, spinal cord injury, trauma/fracture, scoliosis, or cervical deformity were excluded. Additionally, studies looking solely at anterior/posterior cervical fusion, anterior cervical corpectomy and fusion (ACCF), or cervical disc replacement were excluded.

### Information Sources and Search

The literature search was conducted in PubMed and Embase on August 18, 2020. MeSH terms used in the search were herniation; neck; discectomy; total disc replacement; hybridization; surgery; surgical procedures, operative; general surgery; arthroplasty; intervertebral disc; bone matrix; bone transplantation; autologous graft; ilium; allografts; bone marrow; range of motion, articular. Additional keywords used were anterior cervical discectomy and fusion; ACDF; total disc replacement; TDR; hybrid surgery; osteobiologics and radiographic assessment. Limits applied to the search were human, English or Dutch language, full text, and publication year from 01-01-2000. The detailed search strategy is described in Supplementary Appendix II.

### Study Selection

Retrieved articles were screened on title/abstract and subsequently the full text of eligible articles was obtained and screened. This was performed by two reviewers (TH, PW) independently. In case of inconsistency between the two reviewers, a third reviewer (CA) was used to reach consensus. References of full text articles were screened for supplementary studies.

### Data Collection Process and Data Items

Data was extracted using a standardized excel format. Two independent reviewers (TH, PW) obtained general characteristics and research specific data of the included studies. The following general characteristics were collected: first author, publication year, country, study design, hybrid construct, surgical control, number of affected levels, number of operated levels, disc/cage, biologic, sample size, diagnosis, gender, mean age, and length of follow-up as well as information on funding and conflict of interest. The following radiological and clinical outcomes were collected: cervical ROM, fusion rate, ASD, postoperative complications, visual analogue scale (VAS) score, neck disability index (NDI) and Odom criteria.

### Risk of Bias

Risk of bias was assessed by two independent reviewers (TH, PW) using the methodological index for non-randomized studies (MINORS) tool and the risk of bias (RoB 2.0) assessment tool.^15,16^ The global ideal score for the MINORS is 24 for comparative studies and 16 for non-comparative studies. The scores obtained with the MINORS tool can further be interpreted in the following manner for comparative studies: 0-6: very low quality; 7-12: low quality; 13-18: moderate quality and 19-24: high quality and for non-comparative studies: 0-4: very low quality; 5-8: low quality; 9-12: moderate quality and 13-16: high quality.^17,18^ Risk of bias across studies was assessed using the Grading of Recommendations, Assessment, Development and Evaluations (GRADE) approach independently by two reviewers. A third reviewer (CA) was consulted to reach consensus in case of inconsistency between the two reviewers.

## Results

### Study Selection

**Figure 1.**
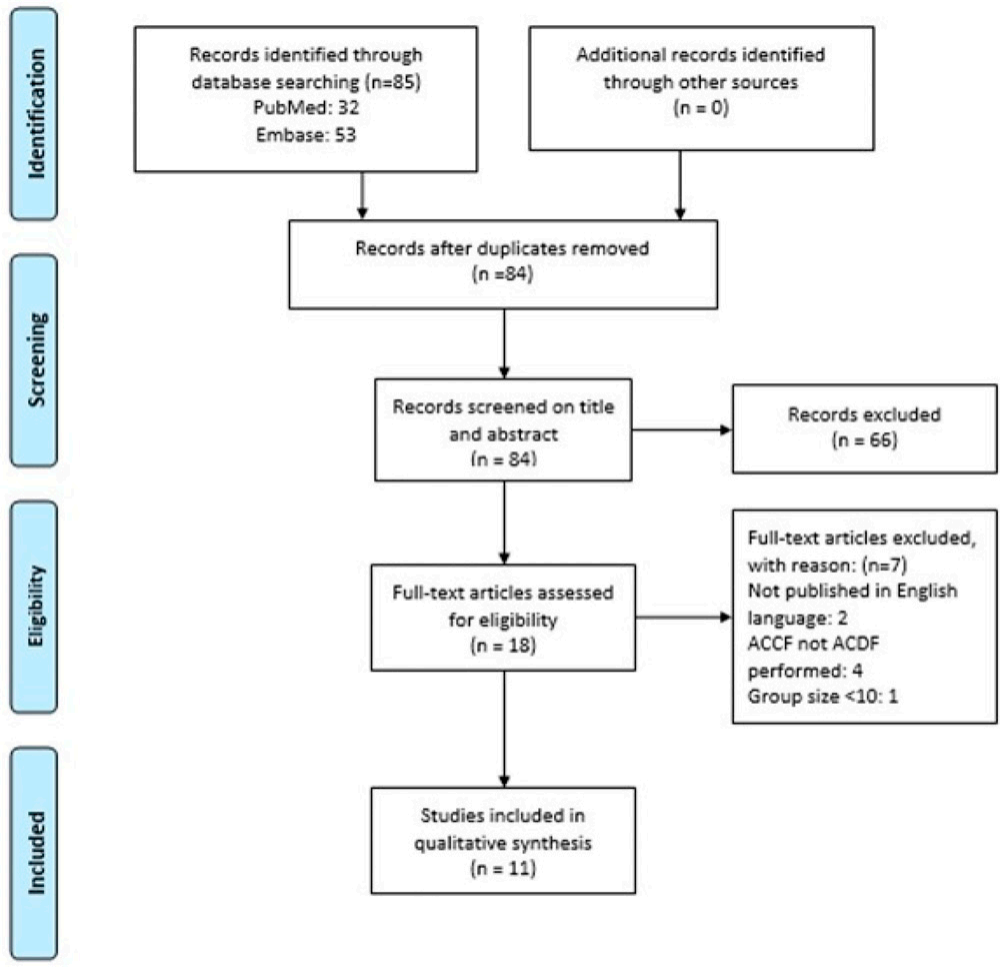
PRISMA flowchart study inclusion. Abbreviations: ACCF, anterior cervical corpectomy and fusion; ACDF, anterior cervical discectomy and fusion.

85 articles were retrieved during the initial search. After removal of duplicates, 84 articles were screened on title and abstract. Consequently, 66 articles were excluded from further analysis. After assessing the full text articles on eligibility and screening the references for additional studies, 11 articles were included in the study. (Figure 1)

### Study Characteristics

One RCT and 10 observational studies were included.^13,19-28^ All studies were published between 2009 and 2020. The majority of the studies (8/11) were conducted in Asia, namely five in China and three in South Korea. The remaining three studies were conducted in Italy, Germany and USA, respectively. The intervention groups of the studies consisted of patients undergoing hybrid surgery. The control groups varied among the studies and were either stand-alone ACDF or TDR. Three of the included studies were non-comparative. The characteristics of the included studies is described in Table 1.

**Table 1.** Characteristics of Included Studies.

Non-comparative studies							
Author	Year	Country	Study Design	Number of Patients (N)		Follow-Up (months)	
Barbagallo et al^13^	2009	Italy	NCS	24		23.8	
Cardoso et al^19^	2011	USA	NCS	31		18.0	
Shi et al^20^	2015	China	NCS	36		41.1	
Comparative studies (HS versus ACDF or TDR)				I	C	I	C
Brotzki et al^21^	2020	Germany	PCS	I1: 15	56	I1:17.7	23.7
				I2: 17		I2: 8.9	
Jang et al^22^	2017	South Korea	RCS	19	30	30.5	32.3
Xu et al^23^	2020	China	RCS	I1: 14	C1: 28	I1: 91.0	C1: 89.4
				I2: 36	C2: 32	I2: 93.3	C2: 89.3
Ji et al^24^	2017	South Korea	PCS	20	20		
Kang et al^25^	2013	China	RCT	12	12	32.8	33.2
Shin et al^26^	2009	South Korea	PCS	20	20	24	24
Wu et al^27^	2017	China	RCS	29	26	32.1	41.6
Xiong et al^28^	2020	China	RCS	48	43	28.9	29.8

Abbreviations: RCT, Randomized Controlled Trial; PCS, Prospective Comparative Study; RCS, Retrospective Comparative Study; NCS, Non-Comparative study; I, intervention group; C, control group.

The selected studies included a total of 588 patients. All comparative studies included sample sizes of more than 10 participants per group and the included prospective case series had sample sizes of above 20 participants. The follow-up period ranged from 8.9 to 93.3 months in the included studies. On average, studies had a minimal follow-up time of 24 months which was deemed as adequate to determine the outcomes of interest.

### Osteobiologics

In four studies the use of osteobiologics as part of the surgical intervention was not reported.^20-23^ Authors were contacted for more information, but no additional information was obtained. The type of osteobiologic that was used varied among the studies, but the most common type among the control groups was an autologous iliac crest graft. DBM and beta-tricalcium phosphate as well as allogeneic bone were additionally found within the control groups. The type of osteobiologic within the intervention groups varied widely eg, DBM, BMP-2, bovine bone, beta-tricalcium phosphate, allogeneic bone and autologous graft. The surgical construct used by the included studies is described in Supplementary Appendix III.

## Qualitative Review

A qualitative description of the radiological and clinical outcomes are presented in Supplementary Appendix IV and V. The included studies prohibited quantitative assessment of the effect of osteobiologics in hybrid surgery since the methodological design and the heterogeneity of the studies did not allow for direct assessment of the research question.

### Radiological Outcomes

Differences were seen in the direction and significance of change when looking at the cervical ROM (C2-C7). Studies mostly reported decreased cervical ROM postoperatively relative to preoperative measurements for both the hybrid surgery and ACDF groups.^19,20,22,23^ Jang et al observed a significant difference between the ROM between the hybrid surgery and ACDF group 6 months postoperatively. The hybrid surgery group showed a faster recovery and reached a ROM closer to the preoperative value. Shi et al reported a significant cervical ROM limitation up to 6 months surgery after surgery. However, this was reversed at last follow-up since the cervical ROM showed a slight but insignificant increase compared to preoperative measurements.

Five studies reported fusion rate in a quantitative manner and two studies qualitatively.^19-22,24,27,28^ Studies reporting quantitative fusion rates found rates ranging between 70 and 100%, whereas Shi et al and Brotzki et al reported satisfactory fusion and the presence of fusion in ACDF in a qualitative manner, respectively. Jang et al was the only study to report a statistically significant difference in fusion rate between patients who underwent hybrid surgery compared to ACDF but the use of osteobiologics was not specified.

Six comparative studies assessed ASD.^21-23,25,27,28^ Brotzki et al reported that the hybrid surgery group produced the best results; the increase of ASD on the Kellgren-Lawrence score was smallest and not significant at final follow-up in the hybrid group compared to preoperative scores. This is supported by Jang et al who found that radiographic ASD presented more frequently in the ACDF group compared to the patients undergoing hybrid surgery, and by Kang et al who reported one case of ASD requiring revision surgery in the ACDF group but no cases in the hybrid surgery group. However, Xiong et al and Xu et al reported that there were no statistically significant differences in ASD between the hybrid surgery and control group.

The rate of subsidence was assessed in two studies.^25,28^ Kang et al detected asymptomatic implant subsidence in one control participant who underwent arthrodesis with a cervical plate system and an autogenous iliac-crest graft, but this required no further intervention. Xiong et al found a subsidence rate of 27% in the participants receiving hybrid surgery compared to 37% in participants who underwent multilevel ACDF. However, the subsidence difference between the two groups was not statistically significant.

Postoperative complications included dysphagia, heterotopic ossification and complications related to prosthesis or cages.^13,19-25,27,28^ Jang et al noted that the group receiving hybrid surgery experienced lower incidences of complications compared to the control group and Ji et al indicated that lower heterotopic ossification rates were found in the hybrid surgery group. However, Xiong et al found more complications in the hybrid surgery group compared to the ACDF group.

### Clinical Outcomes

Brotzki et al showed that the hybrid surgery group had the greatest reduction in arm pain VAS score compared to the patients receiving a dynamic cervical implant or ACDF. Jang et al found that there was a significant decrease in arm pain on VAS in both the hybrid and ACDF group but no statistically significant difference between the two groups was noted 24 months after surgery. Similarly, three studies concluded that there were no statistically significant differences in VAS scores between the hybrid surgery and control group.^25,27,28^ On the contrary, Ji et al and Shin et al found that the hybrid surgery group had less postoperative neck pain compared to the control group (*P* < .05).

All ten studies assessing NDI presented improvement compared to preoperative values.^13,20-28^ Brotzki et al showed that improvement was significantly greater in the hybrid group. This was supported by findings from Xu et al, Ji et al and Shin et al. However, Wu et al, Xiong et al and Kang et al found that there were no statistically significant differences in NDI values between the respective intervention and control groups.

Jang et al was the only study to assess the Odom criteria, which is used to determine general clinical outcome after surgery. The authors concluded that there was significant improvement in both groups, but no statistically significant differences between groups (*P* > .05).

### Risk of Bias Assessment

The single RCT study included was assessed in risk of bias using ROB 2.0. The overall risk of bias was evaluated as being high risk according to the ROB 2.0 algorithm due to the methodological problems with randomization, concealment, and blinding. (Table 2)

**Table 2.** RoB 2.0 for Randomized Controlled Trials.

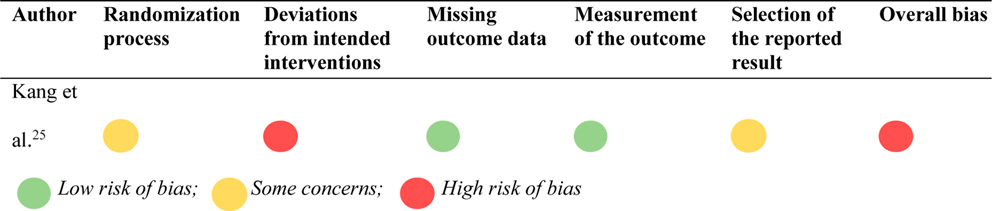

The quality of the included non-randomized studies was described based on the MINORS tool. Most studies suffered from problems with biased endpoint assessments, retrospective collection of data and loss to follow-up. Overall, the studies did not meet the global ideal scores of 24 for comparative and 16 for non-comparative studies. Except for one comparative study, the MINORS scores ranged from 17/24 to 20/24, indicating moderate to high methodological quality. However, one comparative study had low methodological quality with a MINORS score of 11/24. The scores for non-comparative studies were between 7/16 and 11/16, which indicates low to moderate methodological quality. (Table 3)

**Table 3.** MINORS Tool for Non-Randomized Studies.

Author	Clearly Stated Aim	Inclusion of Consecutive Patients	Prospective Collection of Data	Endpoints Appropriate to the Aim of Study	Unbiased Assessment of the Study Endpoint	Follow-Up Period Appropriate to the Aim of Study	Loss to Follow-Up Less Than 5%	Prospective Calculation of the Study Size	An Adequate Control Group	Contemporary Groups	Baseline Equivalence of Groups	Adequate Statistical Analyses	Total Score
Brotzki et al^21^	1	2	2	1	0	1	1	1	1	2	2	2	11/24
Jang et al^22^	2	2	1	2	1	2	0	1	1	2	2	2	18/24
Ji et al^24^	2	2	2	2	1	2	1	1	1	0	1	2	17/24
Shin et al^26^	2	2	1	2	1	2	2	1	1	1	1	2	18/24
Wu et al^27^	2	2	1	2	1	2	0	1	1	2	1	2	17/24
Xiong et al^28^	2	2	1	2	1	2	1	1	2	2	2	2	20/24
Xu et al^23^	2	2	2	2	1	2	0	1	1	2	1	2	18/24
Barbagallo et al^13^	2	1	2	2	2	1	0	1	NA	NA	NA	NA	11/16
Cardoso et al^19^	1	2	1	1	0	1	0	1	NA	NA	NA	NA	7/16
Shi et al^20^	0	2	1	1	1	2	2	1	NA	NA	NA	NA	10/16

MINORS Methodological Index for Non-Randomized studies, NA not available. Items are scored as follows: 0 (not reported); 1 (reported but inadequate); 2 (reported and adequate). Comparative studies: score out of 24; Non-comparative studies: score out of 16.

### Methodological Quality of Evidence

The GRADE procedure used to assess the quality of evidence across studies per outcome could not be performed due to the heterogeneity in studies and consequently the lack of pooled outcomes.

## Discussion

This study investigated the evidence for the use of osteobiologics in multilevel hybrid surgery for cervical DDD. Although studies reported the use of osteobiologics, no studies were identified that directly investigate the role of osteobiologics in hybrid surgery. Rather, studies reporting the use of osteobiologics looked at the effectiveness of hybrid surgery compared to stand-alone ACDF or TDR. Consequently, the heterogeneity in study design, methodological quality and outcome measures prohibited pooling of study results. Therefore, a qualitative review of the radiological and clinical outcomes was presented.

### Radiological/Clinical Outcomes

A decreased cervical ROM was seen in most studies for both the hybrid surgery and the control group.^19,20,22,23^ In general, the fusion rates reported were adequate and only one study reported a statistically significant difference in fusion rates in favour of the hybrid surgery group compared to the ACDF group.^22^ Of the six studies investigating ASD, three studies reported that the hybrid surgery group performed better than the control group.^21,22,25^ The rate of subsidence was lower in the hybrid surgery group, but this was only assessed by two studies.^25,28^ Postoperative complications were noted in varying degrees in both the hybrid surgery and control group.^13,19-28^ Overall, studies reported an improvement in VAS pain scores and NDI values after surgery compared to preoperative scores.^13,20-28^ However, studies varied greatly on whether the reported changes were statistically significant between the hybrid surgery and control group.

### Limitations

This systematic review has several limitations. Firstly, this review is limited by the quality of evidence currently available in the literature. Secondly, no studies could be identified that directly assessed the use of osteobiologics in hybrid surgery. Thus, indirect inferences had to be made, weakening the strength of the evidence found, and prohibiting the pooling of data. Thirdly, language restrictions were applied which potentially could have led to a loss of information considering the majority of included studies were conducted in Asia. Lastly, length of follow-up, study design and outcome measures varied greatly among the included studies, which could explain the heterogeneity in the reported outcomes.

Reduced overall cervical alignment but greater cervical ROM after TDR compared to ACDF has been reported.^29^ Furthermore, literature indicates the importance of mechanical stability and stimulation for osseointegration.^21,30,31^ Considering the differences in physiological movement of the spine when comparing ACDF to TDR, it is assumed that hybrid constructs may lead to altered biomechanical dynamics of the spine as compared to ACDF alone.^21^ This altered biomechanical state may in turn influence bone regeneration and osteobiologic ingrowth.^30^ Thus, to definitively determine the effectiveness of osteobiologics in cervical hybrid surgery, there is a need for high-quality prospective controlled, preferably multicentre randomized trials, with a long-term follow-up (minimum of 24 months) in patients treated by hybrid surgery, comparing different osteobiologics or comparing osteobiologics to autologous bone graft. Additionally, analysis of adjacent level disease, subsidence of the construct and patient-related outcome measures should be included. Furthermore, future studies should focus on the positioning of TDR in the hybrid surgery construct (lower, middle or upper position of the treated levels) and the necessary parameters to indicate the TDR. At present, there is only one randomized controlled trial available of low methodological quality. Additionally, studies should aim to assess endpoints in a blinded manner. Although blinding may be difficult in surgery, outcome assessment on intervention and control patient groups that received surgery can be done by independent assessors that have no prior knowledge of the prescribed treatment and type of osteobiologic used. Furthermore, conformity regarding validated, preferably quantifiable outcome measures is also essential to pool studies in future reviews. Financial aspects related to the use of osteobiologics were beyond the scope of this review. However, there are increased costs related to using biomaterials other than autologous grafts considering material costs as well as personnel costs associated in addition to longer operation times. Thus, research considering the cost-effectiveness of the use of osteobiologics is warranted.

### Conclusions

As of now, there is insufficient clinical evidence to assess the use of osteobiologics in multilevel hybrid surgery. Currently, no studies were identified that directly investigated the role of osteobiologics in hybrid surgery and the available evidence is of low methodological quality. Additionally, there is no consistency in the published results regarding the outcome measures. Hence, there is a need for focused high quality, randomized controlled studies with adequate follow-up time to determine whether the use of osteobiologics is advantageous and cost-effective in hybrid surgery for the treatment of multilevel cervical DDD.

## Supplemental Material

Supplemental Material - The Evidence for the Use of Osteobiologics in Hybrid Constructs (Anterior Cervical Discectomy and Fusion and Total Disc Replacement) in Multilevel Cervical Degenerative Disc Disease: A Systematic Review Supplementary Material for The Evidence for the Use of Osteobiologics in Hybrid Constructs (Anterior Cervical Discectomy and Fusion and Total Disc Replacement) in Multilevel Cervical Degenerative Disc Disease: A Systematic Review by Thomay-Claire A. Hoelen, Paul C. Willems, Arjan Loenen, Hans-Jorg Meisel, Jeffrey Wang, Amit Jain, Zorica Buser, Jacobus J. Arts, and AO Spine Knowledge Forum Degenerative in Global Spine Journal.

## References

[1] He J Liu H Wu T , et al. Association between anterior bone loss and anterior heterotopic ossification in hybrid surgery. BMC Muscoskel Disord. 2020;21(1):1-9. 10.1186/s12891-020-03664-w PMC7545925 33032562

[2] Gao F Mao T Sun W , et al. An updated meta-analysis comparing artificial cervical disc arthroplasty (CDA) versus anterior cervical discectomy and fusion (ACDF) for the treatment of cervical degenerative disc disease (CDDD). Spine. 2015;40(23):1816-1823. 26571063 10.1097/BRS.0000000000001138

[3] Zou S Gao J Xu B Lu X Han Y Meng H . Anterior cervical discectomy and fusion (ACDF) versus cervical disc arthroplasty (CDA) for two contiguous levels cervical disc degenerative disease: A meta-analysis of randomized controlled trials. Eur Spine J. 2017;26(4):985-997. 27314663 10.1007/s00586-016-4655-5

[4] Jia Z Mo Z Ding F He Q Fan Y Ruan D . Hybrid surgery for multilevel cervical degenerative disc diseases: A systematic review of biomechanical and clinical evidence. Eur Spine J. 2014;23(8):1619-1632. 24908252 10.1007/s00586-014-3389-5

[5] Joaquim AF Riew KD . Multilevel cervical arthroplasty: Current evidence. A systematic review. Neurosurg Focus. 2017;42(2):E4. 10.3171/2016.10.FOCUS16354 28142256

[6] Hu L Wu T Liu H , et al. Influence of fusion on the behavior of adjacent disc arthroplasty in contiguous 2-level hybrid surgery in vivo. World Neurosurg. 2019;132:e929-e940. 31323402 10.1016/j.wneu.2019.07.073

[7] Scott-Young M McEntee L Rathbone E Hing W Nielsen D . Clinical outcomes of cervical hybrid reconstructions: A prospective study. Int J Spine Surg. 2020;14(s2):S57-S66. 32994307 10.14444/7092 PMC7528770

[8] Zhang J Meng F Ding Y , et al. Comprehensive analysis of hybrid surgery and anterior cervical discectomy and fusion in cervical diseases: A meta-analysis. Medicine (Baltim). 2020;99(5):e19055. doi:10.1097/md.0000000000019055. PMC7004776 32000453

[9] Cottrill E Pennington Z Lankipalle N , et al. The effect of bioactive glasses on spinal fusion: A cross-disciplinary systematic review and meta-analysis of the preclinical and clinical data. J Clin Neurosci. 2020;78:34-46. doi:10.1016/j.jocn.2020.04.035. 32331941

[10] Giannoudis P Arts JC Schmidmaier G Larsson S . What should be the characteristics of the ideal bone graft substitute? Injury. 2011;42:S1-S2. 10.1016/j.injury.2011.06.001 21700284

[11] Agarwal R Williams K Umscheid CA Welch WC . Osteoinductive bone graft substitutes for lumbar fusion: A systematic review. J Neurosurg Spine. 2009;11(6):729-740. doi:10.3171/2009.6.Spine08669. 19951027

[12] Fischer CR Cassilly R Cantor W Edusei E Hammouri Q Errico T . A systematic review of comparative studies on bone graft alternatives for common spine fusion procedures. Eur Spine J. 2013;22(6):1423-1435. 23440339 10.1007/s00586-013-2718-4 PMC3676568

[13] Barbagallo GM Assietti R Corbino L , et al. Early results and review of the literature of a novel hybrid surgical technique combining cervical arthrodesis and disc arthroplasty for treating multilevel degenerative disc disease: Opposite or complementary techniques? Eur Spine J. 2009;18(suppl 1):29-39. doi:10.1007/s00586-009-0978-9. 19415346 PMC2899598

[14] Moher D Liberati A Tetzlaff J Altman DG Group P . Preferred reporting items for systematic reviews and meta-analyses: The PRISMA statement. PLoS Med. 2009;6(7):e1000097. 19621072 10.1371/journal.pmed.1000097 PMC2707599

[15] Slim K Nini E Forestier D Kwiatkowski F Panis Y Chipponi J . Methodological index for non randomized studies (MINORS): Development and validation of a new instrument. ANZ J Surg. 2003;73(9):712-716. 12956787 10.1046/j.1445-2197.2003.02748.x

[16] Sterne JA Savović J Page MJ , et al. RoB 2: A revised tool for assessing risk of bias in randomised trials. BMJ. 2019;366:l4898. 31462531 10.1136/bmj.l4898

[17] Khan W Khan M Alradwan H Williams R Simunovic N Ayeni OR . Utility of intra-articular hip injections for femoroacetabular impingement: A systematic review. Orthop J Sports Med. 2015;3(9):2325967115601030. 26535395 10.1177/2325967115601030 PMC4622294

[18] Öhlin A Karlsson L Senorski EH , et al. Quality assessment of prospective cohort studies evaluating arthroscopic treatment for femoroacetabular impingement syndrome: A systematic review. Orthop J Sports Med. 2019;7(5):2325967119838533. 31106220 10.1177/2325967119838533 PMC6509989

[19] Cardoso MJ Mendelsohn A Rosner MK . Cervical hybrid arthroplasty with 2 unique fusion techniques. J Neurosurg Spine. 2011;15(1):48-54. doi:10.3171/2011.3.Spine10385. 21456894

[20] Shi J-S Lin B Xue C Zhang H-S Chen Z-D Zhao Z-S . Clinical and radiological outcomes following hybrid surgery in the treatment of multi-level cervical spondylosis: Over a 2-year follow-up. J Orthop Surg Res. 2015;10(1):1-6. 26684799 10.1186/s13018-015-0330-5 PMC4683912

[21] Brotzki C Petridis AK Steiger H-J Bostelmann T Bostelmann R . Comparison of different hybrid techniques for the treatment of multilevel cervical degenerative disc disease–Analysis of prospectively collected clinical, radiologic, and psychological parameters. World Neurosurg. 2020;140:e112-e120. 32371075 10.1016/j.wneu.2020.04.182

[22] Jang S-R Lee S-B Cho K-S . A comparison of anterior cervical discectomy and fusion versus fusion combined with artificial disc replacement for treating 3-level cervical spondylotic disease. J Korean Neurosurg Soc. 2017;60(6):676-683. 29142627 10.3340/jkns.2016.1010.013 PMC5678057

[23] Xu S Liang Y Wang J Yu G Zhu Z Liu H . Cervical spine balance of multilevel total disc replacement, hybrid surgery, and anterior cervical discectomy and fusion with a long-term follow-up. Spine. 2020;45(16):E989-E998. 32706562 10.1097/BRS.0000000000003474

[24] Ji GY Oh CH Shin DA , et al. Artificial disk replacement combined with fusion versus 2-level fusion in cervical 2-level disk disease with a 5-year follow-up. Clin Spine Surg. 2017;30(5):E620-E627. 28525488 10.1097/BSD.0000000000000316

[25] Kang L Lin D Ding Z Liang B Lian K . Artificial disk replacement combined with midlevel ACDF versus multilevel fusion for cervical disk disease involving 3 levels. Orthopedics. 2013;36(1):e88-e94. doi:10.3928/01477447-20121217-24. 23276359

[26] Shin DA Yi S Kim KN Shin HC . Artificial disc replacement combined with fusion versus two-level fusion in cervical two-level disc disease. Spine. 2009;34(11):1153-1159. 19444062 10.1097/BRS.0b013e31819c9d39

[27] Wu T-k Wang B-y Deng M-d , et al. A comparison of anterior cervical discectomy and fusion combined with cervical disc arthroplasty and cervical disc arthroplasty for the treatment of skip-level cervical degenerative disc disease: A retrospective study. Medicine. 2017;96(41):e8112. 29019878 10.1097/MD.0000000000008112 PMC5662301

[28] Xiong Y Yang YD Yu X , et al. Comparison of 2-year follow-up results of the hybrid surgery using Mobi-C combined with ROI-C and anterior cervical discectomy and fusion for the treatment of contiguous two-level cervical degenerative disc diseases. J Clin Neurosci. 2020;73:42-47. doi:10.1016/j.jocn.2020.01.090. 32029368

[29] Di Martino A Papalia R Albo E Cortesi L Denaro L Denaro V . Cervical spine alignment in disc arthroplasty: Should we change our perspective? Eur Spine J. 2015;24(7):810-825. 10.1007/s00586-015-4258-6 26441258

[30] Huang H Liu J Wang L Fan Y . A critical review on the biomechanical study of cervical interbody fusion cage. Med Novel Technol Device. 2021;11:100070.

[31] Shen Y-W Yang Y Liu H , et al. Biomechanical evaluation of intervertebral fusion process after anterior cervical discectomy and fusion: A finite element study. Front Bioeng Biotechnol. 2022;10:842382. 35372323 10.3389/fbioe.2022.842382 PMC8969047

