# Supplementary material for: The Evidence for the Use of Osteobiologics in Hybrid Constructs (Anterior Cervical Discectomy and Fusion and Total Disc Replacement) in Multilevel Cervical Degenerative Disc Disease: A Systematic Review
Source: Global Spine J. 2024 Feb 29;14(2 Suppl):120–8. doi: 10.1177/21925682221150795 (PMC10913915; doi:10.1177/21925682221150795)
Supplement: Supplemental Material - The Evidence for the Use of Osteobiologics in Hybrid Constructs (Anterior Cervical Discectomy and Fusion and Total Disc Replacement) in Multilevel Cervical Degenerative Disc Disease: A Systematic Review [file sj-pdf-1-gsj-10.1177_21925682221150795.pdf]

Supplemental Material for: The evidence for the use of osteobiologics in hybrid constructs (Anterior Cervical Discectomy and Fusion (ACDF) and total disc replacement (TDR)) in multilevel cervical degenerative disc disease: a systematic review

Short title: The use of osteobiologics in hybrid constructs in multilevel cervical degenerative disc disease.

Appendix I

PRISMA 2020 Main Checklist

Topic	No.	Item	Location where item is reported
**TITLE**			
**Title**	1	Identify the report as a systematic review.	Page 1, Line 1
**ABSTRACT**			
**Abstract**	2	See the PRISMA 2020 for Abstracts checklist	
**INTRODUCTION**			
**Rationale**	3	Describe the rationale for the review in the context of existing knowledge.	Section 1, Paragraph 4
**Objectives**	4	Provide an explicit statement of the objective(s) or question(s) the review addresses.	Line 59-61
**METHODS**			
**Eligibility criteria**	5	Specify the inclusion and exclusion criteria for the review and how studies were grouped for the syntheses.	Line 68-77
**Information sources**	6	Specify all databases, registers, websites, organisations, reference lists and other sources searched or consulted to identify studies. Specify the date when each source was last searched or consulted.	Line 78-86
**Search strategy**	7	Present the full search strategies for all databases, registers and websites, including any filters and limits used.	Line 78-86
**Selection process**	8	Specify the methods used to decide whether a study met the inclusion criteria of the review, including how many reviewers screened each record and each report retrieved, whether they worked independently, and if applicable, details of automation tools used in the process.	Line 87-92
**Data collection process**	9	Specify the methods used to collect data from reports, including how many reviewers collected data from each report, whether they worked independently, any processes for obtaining or confirming data from study investigators, and if applicable, details of automation tools used in the process.	Line 93-101
**Data items**	10a	List and define all outcomes for which data were sought. Specify whether all results that were compatible with each outcome domain in each study were sought (e.g. for all measures, time points, analyses), and if not, the methods used to decide which results to collect.	Line 95-101
	10b	List and define all other variables for which data were sought (e.g. participant and intervention characteristics, funding sources). Describe any assumptions made about any missing or unclear information.	Line 95-101

Topic	No.	Item	Location where item is reported
**Study risk of bias assessment**	11	Specify the methods used to assess risk of bias in the included studies, including details of the tool(s) used, how many reviewers assessed each study and whether they worked independently, and if applicable, details of automation tools used in the process.	Line 102-113
**Effect measures**	12	Specify for each outcome the effect measure(s) (e.g. risk ratio, mean difference) used in the synthesis or presentation of results.	/
**Synthesis methods**	13a	Describe the processes used to decide which studies were eligible for each synthesis (e.g. tabulating the study intervention characteristics and comparing against the planned groups for each synthesis (item 5)).	/
	13b	Describe any methods required to prepare the data for presentation or synthesis, such as handling of missing summary statistics, or data conversions.	/
	13c	Describe any methods used to tabulate or visually display results of individual studies and syntheses.	/
	13d	Describe any methods used to synthesize results and provide a rationale for the choice(s). If meta-analysis was performed, describe the model(s), method(s) to identify the presence and extent of statistical heterogeneity, and software package(s) used.	/
	13e	Describe any methods used to explore possible causes of heterogeneity among study results (e.g. subgroup analysis, meta-regression).	/
	13f	Describe any sensitivity analyses conducted to assess robustness of the synthesized results.	/
**Reporting bias assessment**	14	Describe any methods used to assess risk of bias due to missing results in a synthesis (arising from reporting biases).	/
**Certainty assessment**	15	Describe any methods used to assess certainty (or confidence) in the body of evidence for an outcome.	110-113
**RESULTS**			
**Study selection**	16a	Describe the results of the search and selection process, from the number of records identified in the search to the number of studies included in the review, ideally using a flow diagram.	Line 115-119
	16b	Cite studies that might appear to meet the inclusion criteria, but which were excluded, and explain why they were excluded.	Line 115-119
**Study characteristics**	17	Cite each included study and present its characteristics.	Line 120-133
**Risk of bias in studies**	18	Present assessments of risk of bias for each included study.	Line 198-217
**Results of individual studies**	19	For all outcomes, present, for each study: (a) summary statistics for each group (where appropriate) and (b) an effect estimate and its precision (e.g. confidence/credible interval), ideally using structured tables or plots.	Line 142-197
**Results of syntheses**	20a	For each synthesis, briefly summarise the characteristics and risk of bias among contributing studies.	Line 142-197

Topic	No.	Item	Location where item is reported
	20b	Present results of all statistical syntheses conducted. If meta-analysis was done, present for each the summary estimate and its precision (e.g. confidence/credible interval) and measures of statistical heterogeneity. If comparing groups, describe the direction of the effect.	/
	20c	Present results of all investigations of possible causes of heterogeneity among study results.	/
	20d	Present results of all sensitivity analyses conducted to assess the robustness of the synthesized results.	/
**Reporting biases**	21	Present assessments of risk of bias due to missing results (arising from reporting biases) for each synthesis assessed.	/
**Certainty of evidence**	22	Present assessments of certainty (or confidence) in the body of evidence for each outcome assessed.	210-218
**DISCUSSION**			
**Discussion**	23a	Provide a general interpretation of the results in the context of other evidence.	Line 218-236
	23b	Discuss any limitations of the evidence included in the review.	Line 237-263
	23c	Discuss any limitations of the review processes used.	Line 237-263
	23d	Discuss implications of the results for practice, policy, and future research.	Line 264-271
**OTHER INFORMATION**			
**Registration and protocol**	24a	Provide registration information for the review, including register name and registration number, or state that the review was not registered.	Line 63-67
	24b	Indicate where the review protocol can be accessed, or state that a protocol was not prepared.	Line 64-65
	24c	Describe and explain any amendments to information provided at registration or in the protocol.	/
**Support**	25	Describe sources of financial or non-financial support for the review, and the role of the funders or sponsors in the review.	Line 275-277
**Competing interests**	26	Declare any competing interests of review authors.	Line 278-279
**Availability of data, code and other materials**	27	Report which of the following are publicly available and where they can be found: template data collection forms; data extracted from included studies; data used for all analyses; analytic code; any other materials used in the review.	Supplementary Material

PRIMSA Abstract Checklist

Topic	No.	Item	Reported?
**TITLE**			
**Title**	1	Identify the report as a systematic review.	Yes
**BACKGROUND**			
**Objectives**	2	Provide an explicit statement of the main objective(s) or question(s) the review addresses.	Yes
**METHODS**			
**Eligibility criteria**	3	Specify the inclusion and exclusion criteria for the review.	Yes
**Information sources**	4	Specify the information sources (e.g. databases, registers) used to identify studies and the date when each was last searched.	Yes
**Risk of bias**	5	Specify the methods used to assess risk of bias in the included studies.	Yes
**Synthesis of results**	6	Specify the methods used to present and synthesize results.	Yes
**RESULTS**			
**Included studies**	7	Give the total number of included studies and participants and summarise relevant characteristics of studies.	Yes
**Synthesis of results**	8	Present results for main outcomes, preferably indicating the number of included studies and participants for each. If meta-analysis was done, report the summary estimate and confidence/credible interval. If comparing groups, indicate the direction of the effect (i.e. which group is favoured).	Yes
**DISCUSSION**			
**Limitations of evidence**	9	Provide a brief summary of the limitations of the evidence included in the review (e.g. study risk of bias, inconsistency and imprecision).	Yes
**Interpretation**	10	Provide a general interpretation of the results and important implications.	Yes
**OTHER**			
**Funding**	11	Specify the primary source of funding for the review.	Yes
**Registration**	12	Provide the register name and registration number.	Yes

From: Page MJ, McKenzie JE, Bossuyt PM, Boutron I, Hoffmann TC, Mulrow CD, et al. The PRISMA 2020 statement: an updated guideline for reporting systematic reviews. MetaArXiv. 2020, September 14. DOI: 10.31222/osf.io/v7gm2. For more information, visit: www.prisma-statement.org

16 Appendix II

**Search Topic**	**#**	**Search Terms**	**# of records**
**Population:** Adult patients (18-80), herniated or degenerative cervical discs	#1	"hernia"[MeSH Terms] OR "hernia"[All Fields] OR "herniation"[All Fields] OR "herniations"[All Fields] OR "herniate"[All Fields] OR "herniated"[All Fields] OR "herniates"[All Fields] OR "herniating"[All Fields] OR (("degenerative"[All Fields] OR "degeneratively"[All Fields] OR "degeneratives"[All Fields]) AND ("cervic"[All Fields] OR "cervicals"[All Fields] OR "cervices"[All Fields] OR "neck"[MeSH Terms] OR "neck"[All Fields] OR "cervical"[All Fields] OR "uterine cervicitis"[MeSH Terms] OR ("uterine"[All Fields] AND "cervicitis"[All Fields]) OR "uterine cervicitis"[All Fields] OR "cervicitis"[All Fields]) AND ("neck"[MeSH Terms] OR "neck"[All Fields]) AND "disc"[All Fields])	102,987
	#2	(("hernia"[MeSH Terms] OR "hernia"[All Fields] OR "herniation"[All Fields] OR "herniations"[All Fields] OR "herniate"[All Fields] OR "herniated"[All Fields] OR "herniates"[All Fields] OR "herniating"[All Fields]) AND ("cervic"[All Fields] OR "cervicals"[All Fields] OR "cervices"[All Fields] OR "neck"[MeSH Terms] OR "neck"[All Fields] OR "cervical"[All Fields] OR "uterine cervicitis"[MeSH Terms] OR ("uterine"[All Fields] AND "cervicitis"[All Fields]) OR "uterine cervicitis"[All Fields] OR "cervicitis"[All Fields]) AND ("neck"[MeSH Terms] OR "neck"[All Fields]) AND "disc"[All Fields]) OR (("degenerative"[All Fields] OR "degeneratively"[All Fields] OR "degeneratives"[All Fields]) AND ("cervic"[All Fields] OR "cervicals"[All Fields] OR "cervices"[All Fields] OR "neck"[MeSH Terms] OR "neck"[All Fields] OR "cervical"[All Fields] OR "uterine cervicitis"[MeSH Terms] OR ("uterine"[All Fields] AND "cervicitis"[All Fields]) OR "uterine cervicitis"[All Fields] OR "cervicitis"[All Fields]) AND ("neck"[MeSH Terms] OR "neck"[All Fields]) AND "disc"[All Fields])	1,440
	#3	("Disc"[All Fields] OR "Disk"[All Fields]) AND ("degenerative"[All Fields] OR "degeneratively"[All Fields] OR "degeneratives"[All Fields] OR ("degenerate"[All Fields] OR "degenerated"[All Fields] OR "degenerately"[All Fields] OR "degenerates"[All Fields] OR "degenerating"[All Fields] OR "degeneration"[All Fields] OR "degenerations"[All Fields]) OR ("degenerate"[All Fields] OR "degenerated"[All Fields] OR "degenerately"[All Fields] OR "degenerates"[All Fields] OR "degenerating"[All Fields] OR "degeneration"[All Fields] OR "degenerations"[All Fields]) OR "displacement*"[All Fields] OR "herniation*"[All Fields])	36,268
	#4	#1 OR #2 OR #3	116,415
**Intervention:** Multilevel, 2-4 levels ACDF or ACDF and TDR	#5	"Anterior Cervical Discectomy and Fusion"[All Fields] OR "Anterior Cervical Diskectomy And Fusion"[All Fields] OR "ACDF"[All Fields]	2367
	#6	"Diskectomy"[MeSH Terms] OR ("Diskectomy"[MeSH Terms] OR "Diskectomy"[All Fields] OR "discectomies"[All Fields] OR "discectomy"[All Fields]) OR ("Diskectomy"[MeSH Terms] OR "Diskectomy"[All Fields] OR "diskectomies"[All Fields]) OR ("total disc replacement"[MeSH Terms] OR ("total"[All Fields] AND "disc"[All Fields] AND "replacement"[All Fields]) OR "total disc replacement"[All Fields])	10,708
	#7	#5 AND #6	2,184
	#8	("anterior"[All Fields] OR "anteriores"[All Fields] OR "anteriorization"[All Fields] OR "anteriorized"[All Fields] OR "anteriors"[All Fields]) AND ("cervic"[All Fields] OR "cervicals"[All Fields] OR "cervices"[All Fields] OR "neck"[MeSH Terms] OR "neck"[All Fields] OR "cervical"[All Fields] OR "uterine cervicitis"[MeSH Terms] OR ("uterine"[All Fields] AND "cervicitis"[All Fields]) OR "uterine cervicitis"[All Fields] OR "cervicitis"[All Fields]) AND ("diskectomy"[MeSH Terms] OR "diskectomy"[All Fields] OR "discectomies"[All Fields] OR "discectomy"[All Fields]) AND ("fusion"[All Fields] OR "fusions"[All Fields]) AND ("combinable"[All Fields] OR "combinated"[All Fields] OR "combination"[All Fields] OR "combinational"[All Fields] OR "combinations"[All Fields] OR "combinative"[All Fields] OR "combine"[All Fields] OR "combined"[All Fields] OR "combines"[All Fields] OR "combining"[All Fields]) AND ("total disc replacement"[MeSH Terms] OR ("total"[All Fields] AND "disc"[All Fields] AND "replacement"[All Fields]) OR "total disc replacement"[All Fields])	23
	#9	"ACDF"[All Fields] AND ("combinable"[All Fields] OR "combinated"[All Fields] OR "combination"[All Fields] OR "combinational"[All Fields] OR "combinations"[All Fields] OR "combinative"[All Fields] OR "combine"[All Fields] OR "combined"[All Fields] OR "combines"[All Fields] OR "combining"[All Fields]) AND "TDR"[All Fields]	5
	#10	("chimera"[MeSH Terms] OR "chimera"[All Fields] OR "hybrid"[All Fields] OR	13

		"hybrids"[All Fields] OR "hybrid s"[All Fields] OR "hybridation"[All Fields] OR "hybridisations"[All Fields] OR "hybridise"[All Fields] OR "hybridised"[All Fields] OR "hybridises"[All Fields] OR "hybridising"[All Fields] OR "hybridity"[All Fields] OR "hybridization, genetic"[MeSH Terms] OR ("hybridization"[All Fields] AND "genetic"[All Fields]) OR "genetic hybridization"[All Fields] OR "hybridisation"[All Fields] OR "hybridizations"[All Fields] OR "hybridize"[All Fields] OR "hybridized"[All Fields] OR "hybridizes"[All Fields] OR "hybridizing"[All Fields] OR "nucleic acid hybridization"[MeSH Terms] OR ("nucleic"[All Fields] AND "acid"[All Fields] AND "hybridization"[All Fields]) OR "nucleic acid hybridization"[All Fields] OR "hybridization"[All Fields]) AND ("surgery"[MeSH Subheading] OR "surgery"[All Fields] OR "surgical procedures, operative"[MeSH Terms] OR ("surgical"[All Fields] AND "procedures"[All Fields] AND "operative"[All Fields]) OR "operative surgical procedures"[All Fields] OR "general surgery"[MeSH Terms] OR ("general"[All Fields] AND "surgery"[All Fields]) OR "general surgery"[All Fields] OR "surgery s"[All Fields] OR "surgerys"[All Fields] OR "surgeries"[All Fields]) AND ("anterior"[All Fields] OR "anteriores"[All Fields] OR "anteriorization"[All Fields] OR "anteriorized"[All Fields] OR "anteriors"[All Fields]) AND ("cervic"[All Fields] OR "cervicals"[All Fields] OR "cervices"[All Fields] OR "neck"[MeSH Terms] OR "neck"[All Fields] OR "cervical"[All Fields] OR "uterine cervicitis"[MeSH Terms] OR ("uterine"[All Fields] AND "cervicitis"[All Fields]) OR "uterine cervicitis"[All Fields] OR "cervicitis"[All Fields]) AND ("diskectomy"[MeSH Terms] OR "diskectomy"[All Fields] OR "discectomies"[All Fields] OR "discectomy"[All Fields]) AND ("fusion"[All Fields] OR "fusions"[All Fields]) AND ("combinable"[All Fields] OR "combinated"[All Fields] OR "combination"[All Fields] OR "combinational"[All Fields] OR "combinations"[All Fields] OR "combinative"[All Fields] OR "combine"[All Fields] OR "combined"[All Fields] OR "combines"[All Fields] OR "combining"[All Fields]) AND ("total disc replacement"[MeSH Terms] OR ("total"[All Fields] AND "disc"[All Fields] AND "replacement"[All Fields]) OR "total disc replacement"[All Fields])	
	#11	("chimera"[MeSH Terms] OR "chimera"[All Fields] OR "hybrid"[All Fields] OR "hybrids"[All Fields] OR "hybrid s"[All Fields] OR "hybridation"[All Fields] OR "hybridisations"[All Fields] OR "hybridise"[All Fields] OR "hybridised"[All Fields] OR "hybridises"[All Fields] OR "hybridising"[All Fields] OR "hybridity"[All Fields] OR "hybridization, genetic"[MeSH Terms] OR ("hybridization"[All Fields] AND "genetic"[All Fields]) OR "genetic hybridization"[All Fields] OR "hybridisation"[All Fields] OR "hybridizations"[All Fields] OR "hybridize"[All Fields] OR "hybridized"[All Fields] OR "hybridizes"[All Fields] OR "hybridizing"[All Fields] OR "nucleic acid hybridization"[MeSH Terms] OR ("nucleic"[All Fields] AND "acid"[All Fields] AND "hybridization"[All Fields]) OR "nucleic acid hybridization"[All Fields] OR "hybridization"[All Fields]) AND ("surgery"[MeSH Subheading] OR "surgery"[All Fields] OR "surgical procedures, operative"[MeSH Terms] OR ("surgical"[All Fields] AND "procedures"[All Fields] AND "operative"[All Fields]) OR "operative surgical procedures"[All Fields] OR "general surgery"[MeSH Terms] OR ("general"[All Fields] AND "surgery"[All Fields]) OR "general surgery"[All Fields] OR "surgery s"[All Fields] OR "surgerys"[All Fields] OR "surgeries"[All Fields]) AND ("anterior"[All Fields] OR "anteriores"[All Fields] OR "anteriorization"[All Fields] OR "anteriorized"[All Fields] OR "anteriors"[All Fields]) AND ("cervic"[All Fields] OR "cervicals"[All Fields] OR "cervices"[All Fields] OR "neck"[MeSH Terms] OR "neck"[All Fields] OR "cervical"[All Fields] OR "uterine cervicitis"[MeSH Terms] OR ("uterine"[All Fields] AND "cervicitis"[All Fields]) OR "uterine cervicitis"[All Fields] OR "cervicitis"[All Fields]) AND ("diskectomy"[MeSH Terms] OR "diskectomy"[All Fields] OR "discectomies"[All Fields] OR "discectomy"[All Fields]) AND ("fusion"[All Fields] OR "fusions"[All Fields]) AND ("combinable"[All Fields] OR "combinated"[All Fields] OR "combination"[All Fields] OR "combinational"[All Fields] OR "combinations"[All Fields] OR "combinative"[All Fields] OR "combine"[All Fields] OR "combined"[All Fields] OR "combines"[All Fields] OR "combining"[All Fields]) AND ("total disc replacement"[MeSH Terms] OR ("total"[All Fields] AND "disc"[All Fields] AND "replacement"[All Fields]) OR "total disc replacement"[All Fields]) AND ("multilevel"[All Fields] OR "multilevels"[All Fields])	7
	#12	("chimera"[MeSH Terms] OR "chimera"[All Fields] OR "hybrid"[All Fields] OR "hybrids"[All Fields] OR "hybrid s"[All Fields] OR "hybridation"[All Fields] OR "hybridisations"[All Fields] OR "hybridise"[All Fields] OR "hybridised"[All Fields] OR "hybridises"[All Fields] OR "hybridising"[All Fields] OR "hybridity"[All Fields] OR "hybridization, genetic"[MeSH Terms] OR ("hybridization"[All Fields] AND "genetic"[All Fields]) OR "genetic hybridization"[All Fields] OR "hybridisation"[All Fields] OR "hybridizations"[All Fields] OR "hybridize"[All Fields] OR "hybridized"[All Fields] OR "hybridizes"[All Fields] OR "hybridizing"[All Fields] OR "nucleic acid hybridization"[MeSH Terms] OR ("nucleic"[All Fields] AND "acid"[All	18

		Fields] AND "hybridization"[All Fields]) OR "nucleic acid hybridization"[All Fields] OR "hybridization"[All Fields]) AND ("surgery"[MeSH Subheading] OR "surgery"[All Fields] OR "surgical procedures, operative"[MeSH Terms] OR ("surgical"[All Fields] AND "procedures"[All Fields] AND "operative"[All Fields]) OR "operative surgical procedures"[All Fields] OR "general surgery"[MeSH Terms] OR ("general"[All Fields] AND "surgery"[All Fields]) OR "general surgery"[All Fields] OR "surgery s"[All Fields] OR "surgerys"[All Fields] OR "surgeries"[All Fields]) AND ("anterior"[All Fields] OR "anteriores"[All Fields] OR "anteriorization"[All Fields] OR "anteriorized"[All Fields] OR "anteriors"[All Fields]) AND ("cervic"[All Fields] OR "cervicals"[All Fields] OR "cervices"[All Fields] OR "neck"[MeSH Terms] OR "neck"[All Fields] OR "cervical"[All Fields] OR "uterine cervicitis"[MeSH Terms] OR ("uterine"[All Fields] AND "cervicitis"[All Fields]) OR "uterine cervicitis"[All Fields] OR "cervicitis"[All Fields]) AND ("diskectomy"[MeSH Terms] OR "diskectomy"[All Fields] OR "discectomies"[All Fields] OR "discectomy"[All Fields]) AND ("fusion"[All Fields] OR "fusions"[All Fields]) AND ("combinable"[All Fields] OR "combinated"[All Fields] OR "combination"[All Fields] OR "combinational"[All Fields] OR "combinations"[All Fields] OR "combinative"[All Fields] OR "combine"[All Fields] OR "combined"[All Fields] OR "combines"[All Fields] OR "combining"[All Fields]) AND ("total disc replacement"[MeSH Terms] OR ("total"[All Fields] AND "disc"[All Fields] AND "replacement"[All Fields]) OR "total disc replacement"[All Fields] OR ("artificial"[All Fields] AND "disc"[All Fields] AND "replacement"[All Fields]) OR "artificial disc replacement"[All Fields])	
	#13	("anterior"[All Fields] OR "anteriores"[All Fields] OR "anteriorization"[All Fields] OR "anteriorized"[All Fields] OR "anteriors"[All Fields]) AND ("cervic"[All Fields] OR "cervicals"[All Fields] OR "cervices"[All Fields] OR "neck"[MeSH Terms] OR "neck"[All Fields] OR "cervical"[All Fields] OR "uterine cervicitis"[MeSH Terms] OR ("uterine"[All Fields] AND "cervicitis"[All Fields]) OR "uterine cervicitis"[All Fields] OR "cervicitis"[All Fields]) AND ("diskectomy"[MeSH Terms] OR "diskectomy"[All Fields] OR "discectomies"[All Fields] OR "discectomy"[All Fields]) AND ("fusion"[All Fields] OR "fusions"[All Fields]) AND ("combinable"[All Fields] OR "combinated"[All Fields] OR "combination"[All Fields] OR "combinational"[All Fields] OR "combinations"[All Fields] OR "combinative"[All Fields] OR "combine"[All Fields] OR "combined"[All Fields] OR "combines"[All Fields] OR "combining"[All Fields]) AND ("total"[All Fields] OR "totaled"[All Fields] OR "totaling"[All Fields] OR "totalled"[All Fields] OR "totalling"[All Fields] OR "totals"[All Fields]) AND ("intervertebral disc"[MeSH Terms] OR ("intervertebral"[All Fields] AND "disc"[All Fields]) OR "intervertebral disc"[All Fields]) AND ("arthroplasty"[MeSH Terms] OR "arthroplasty"[All Fields] OR "arthroplasties"[All Fields])	14
	#14	("chimera"[MeSH Terms] OR "chimera"[All Fields] OR "hybrid"[All Fields] OR "hybrids"[All Fields] OR "hybrid s"[All Fields] OR "hybridation"[All Fields] OR "hybridisations"[All Fields] OR "hybridise"[All Fields] OR "hybridised"[All Fields] OR "hybridises"[All Fields] OR "hybridising"[All Fields] OR "hybridity"[All Fields] OR "hybridization, genetic"[MeSH Terms] OR ("hybridization"[All Fields] AND "genetic"[All Fields]) OR "genetic hybridization"[All Fields] OR "hybridisation"[All Fields] OR "hybridizations"[All Fields] OR "hybridize"[All Fields] OR "hybridized"[All Fields] OR "hybridizes"[All Fields] OR "hybridizing"[All Fields] OR "nucleic acid hybridization"[MeSH Terms] OR ("nucleic"[All Fields] AND "acid"[All Fields] AND "hybridization"[All Fields]) OR "nucleic acid hybridization"[All Fields] OR "hybridization"[All Fields]) AND ("surgery"[MeSH Subheading] OR "surgery"[All Fields] OR "surgical procedures, operative"[MeSH Terms] OR ("surgical"[All Fields] AND "procedures"[All Fields] AND "operative"[All Fields]) OR "operative surgical procedures"[All Fields] OR "general surgery"[MeSH Terms] OR ("general"[All Fields] AND "surgery"[All Fields]) OR "general surgery"[All Fields] OR "surgery s"[All Fields] OR "surgerys"[All Fields] OR "surgeries"[All Fields]) AND ("anterior"[All Fields] OR "anteriores"[All Fields] OR "anteriorization"[All Fields] OR "anteriorized"[All Fields] OR "anteriors"[All Fields]) AND ("cervic"[All Fields] OR "cervicals"[All Fields] OR "cervices"[All Fields] OR "neck"[MeSH Terms] OR "neck"[All Fields] OR "cervical"[All Fields] OR "uterine cervicitis"[MeSH Terms] OR ("uterine"[All Fields] AND "cervicitis"[All Fields]) OR "uterine cervicitis"[All Fields] OR "cervicitis"[All Fields]) AND ("diskectomy"[MeSH Terms] OR "diskectomy"[All Fields] OR "discectomies"[All Fields] OR "discectomy"[All Fields]) AND ("fusion"[All Fields] OR "fusions"[All Fields]) AND ("combinable"[All Fields] OR "combinated"[All Fields] OR "combination"[All Fields] OR "combinational"[All Fields] OR "combinations"[All Fields] OR "combinative"[All Fields] OR "combine"[All Fields] OR "combined"[All Fields] OR "combines"[All Fields] OR "combining"[All Fields]) AND ("total"[All Fields] OR "totaled"[All Fields] OR "totaling"[All Fields] OR "totalled"[All Fields] OR "totalling"[All Fields] OR "totals"[All Fields]) AND	8

		("intervertebral disc"[MeSH Terms] OR ("intervertebral"[All Fields] AND "disc"[All Fields]) OR "intervertebral disc"[All Fields]) AND ("arthroplasty"[MeSH Terms] OR "arthroplasty"[All Fields] OR "arthroplasties"[All Fields])	
	#15	("anterior"[All Fields] OR "anteriores"[All Fields] OR "anteriorization"[All Fields] OR "anteriorized"[All Fields] OR "anteriors"[All Fields]) AND ("cervic"[All Fields] OR "cervicals"[All Fields] OR "cervices"[All Fields] OR "neck"[MeSH Terms] OR "neck"[All Fields] OR "cervical"[All Fields] OR "uterine cervicitis"[MeSH Terms] OR ("uterine"[All Fields] AND "cervicitis"[All Fields]) OR "uterine cervicitis"[All Fields] OR "cervicitis"[All Fields]) AND ("diskectomy"[MeSH Terms] OR "diskectomy"[All Fields] OR "discectomies"[All Fields] OR "discectomy"[All Fields]) AND ("fusion"[All Fields] OR "fusions"[All Fields]) AND ("combinable"[All Fields] OR "combinated"[All Fields] OR "combination"[All Fields] OR "combinational"[All Fields] OR "combinations"[All Fields] OR "combinative"[All Fields] OR "combine"[All Fields] OR "combined"[All Fields] OR "combines"[All Fields] OR "combining"[All Fields]) AND ("total"[All Fields] OR "totaled"[All Fields] OR "totaling"[All Fields] OR "totalled"[All Fields] OR "totalling"[All Fields] OR "totals"[All Fields]) AND ("intervertebral disc"[MeSH Terms] OR ("intervertebral"[All Fields] AND "disc"[All Fields]) OR "intervertebral disc"[All Fields]) AND ("arthroplasty"[MeSH Terms] OR "arthroplasty"[All Fields] OR "arthroplasties"[All Fields]) AND ("multilevel"[All Fields] OR "multilevels"[All Fields])	5
	#16	("chimera"[MeSH Terms] OR "chimera"[All Fields] OR "hybrid"[All Fields] OR "hybrids"[All Fields] OR "hybrid s"[All Fields] OR "hybridation"[All Fields] OR "hybridisations"[All Fields] OR "hybridise"[All Fields] OR "hybridised"[All Fields] OR "hybridises"[All Fields] OR "hybridising"[All Fields] OR "hybridity"[All Fields] OR "hybridization, genetic"[MeSH Terms] OR ("hybridization"[All Fields] AND "genetic"[All Fields]) OR "genetic hybridization"[All Fields] OR "hybridisation"[All Fields] OR "hybridizations"[All Fields] OR "hybridize"[All Fields] OR "hybridized"[All Fields] OR "hybridizes"[All Fields] OR "hybridizing"[All Fields] OR "nucleic acid hybridization"[MeSH Terms] OR ("nucleic"[All Fields] AND "acid"[All Fields] AND "hybridization"[All Fields]) OR "nucleic acid hybridization"[All Fields] OR "hybridization"[All Fields]) AND ("surgery"[MeSH Subheading] OR "surgery"[All Fields] OR "surgical procedures, operative"[MeSH Terms] OR ("surgical"[All Fields] AND "procedures"[All Fields] AND "operative"[All Fields]) OR "operative surgical procedures"[All Fields] OR "general surgery"[MeSH Terms] OR ("general"[All Fields] AND "surgery"[All Fields]) OR "general surgery"[All Fields] OR "surgery s"[All Fields] OR "surgerys"[All Fields] OR "surgeries"[All Fields]) AND ("anterior"[All Fields] OR "anteriores"[All Fields] OR "anteriorization"[All Fields] OR "anteriorized"[All Fields] OR "anteriors"[All Fields]) AND ("cervic"[All Fields] OR "cervicals"[All Fields] OR "cervices"[All Fields] OR "neck"[MeSH Terms] OR "neck"[All Fields] OR "cervical"[All Fields] OR "uterine cervicitis"[MeSH Terms] OR ("uterine"[All Fields] AND "cervicitis"[All Fields]) OR "uterine cervicitis"[All Fields] OR "cervicitis"[All Fields]) AND ("diskectomy"[MeSH Terms] OR "diskectomy"[All Fields] OR "discectomies"[All Fields] OR "discectomy"[All Fields]) AND ("fusion"[All Fields] OR "fusions"[All Fields]) AND ("combinable"[All Fields] OR "combinated"[All Fields] OR "combination"[All Fields] OR "combinational"[All Fields] OR "combinations"[All Fields] OR "combinative"[All Fields] OR "combine"[All Fields] OR "combined"[All Fields] OR "combines"[All Fields] OR "combining"[All Fields]) AND ("total"[All Fields] OR "totaled"[All Fields] OR "totaling"[All Fields] OR "totalled"[All Fields] OR "totalling"[All Fields] OR "totals"[All Fields]) AND ("intervertebral disc"[MeSH Terms] OR ("intervertebral"[All Fields] AND "disc"[All Fields]) OR "intervertebral disc"[All Fields]) AND ("arthroplasty"[MeSH Terms] OR "arthroplasty"[All Fields] OR "arthroplasties"[All Fields]) AND ("multilevel"[All Fields] OR "multilevels"[All Fields])	5
	#17	("multilevel"[All Fields] OR "multilevels"[All Fields]) AND "ACDF"[All Fields] AND "TDR"[All Fields] AND ("chimera"[MeSH Terms] OR "chimera"[All Fields] OR "hybrid"[All Fields] OR "hybrids"[All Fields] OR "hybrid s"[All Fields] OR "hybridation"[All Fields] OR "hybridisations"[All Fields] OR "hybridise"[All Fields] OR "hybridised"[All Fields] OR "hybridises"[All Fields] OR "hybridising"[All Fields] OR "hybridity"[All Fields] OR "hybridization, genetic"[MeSH Terms] OR ("hybridization"[All Fields] AND "genetic"[All Fields]) OR "genetic hybridization"[All Fields] OR "hybridisation"[All Fields] OR "hybridizations"[All Fields] OR "hybridize"[All Fields] OR "hybridized"[All Fields] OR "hybridizes"[All Fields] OR "hybridizing"[All Fields] OR "nucleic acid hybridization"[MeSH Terms] OR ("nucleic"[All Fields] AND "acid"[All Fields] AND "hybridization"[All Fields]) OR "nucleic acid hybridization"[All Fields] OR "hybridization"[All Fields]) AND ("construct s"[All Fields] OR "constructed"[All Fields] OR "constructing"[All Fields] OR "construction"[All Fields] OR "constructions"[All Fields] OR "constructive"[All	2

		Fields] OR "constructively"[All Fields] OR "constructs"[All Fields] OR "dna, recombinant"[MeSH Terms] OR ("dna"[All Fields] AND "recombinant"[All Fields]) OR "recombinant dna"[All Fields] OR "construct"[All Fields])	
	#18	("chimera"[MeSH Terms] OR "chimera"[All Fields] OR "hybrid"[All Fields] OR "hybrids"[All Fields] OR "hybrid s"[All Fields] OR "hybridation"[All Fields] OR "hybridisations"[All Fields] OR "hybridise"[All Fields] OR "hybridised"[All Fields] OR "hybridises"[All Fields] OR "hybridising"[All Fields] OR "hybridity"[All Fields] OR "hybridization, genetic"[MeSH Terms] OR ("hybridization"[All Fields] AND "genetic"[All Fields]) OR "genetic hybridization"[All Fields] OR "hybridisation"[All Fields] OR "hybridizations"[All Fields] OR "hybridize"[All Fields] OR "hybridized"[All Fields] OR "hybridizes"[All Fields] OR "hybridizing"[All Fields] OR "nucleic acid hybridization"[MeSH Terms] OR ("nucleic"[All Fields] AND "acid"[All Fields] AND "hybridization"[All Fields]) OR "nucleic acid hybridization"[All Fields] OR "hybridization"[All Fields]) AND ("surgery"[MeSH Subheading] OR "surgery"[All Fields] OR "surgical procedures, operative"[MeSH Terms] OR ("surgical"[All Fields] AND "procedures"[All Fields] AND "operative"[All Fields]) OR "operative surgical procedures"[All Fields] OR "general surgery"[MeSH Terms] OR ("general"[All Fields] AND "surgery"[All Fields]) OR "general surgery"[All Fields] OR "surgery s"[All Fields] OR "surgerys"[All Fields] OR "surgeries"[All Fields]) AND ("anterior"[All Fields] OR "anteriores"[All Fields] OR "anteriorization"[All Fields] OR "anteriorized"[All Fields] OR "anteriors"[All Fields]) AND ("cervic"[All Fields] OR "cervicals"[All Fields] OR "cervices"[All Fields] OR "neck"[MeSH Terms] OR "neck"[All Fields] OR "cervical"[All Fields] OR "uterine cervicitis"[MeSH Terms] OR ("uterine"[All Fields] AND "cervicitis"[All Fields]) OR "uterine cervicitis"[All Fields] OR "cervicitis"[All Fields]) AND ("diskectomy"[MeSH Terms] OR "diskectomy"[All Fields] OR "discectomies"[All Fields] OR "discectomy"[All Fields]) AND ("fusion"[All Fields] OR "fusions"[All Fields]) AND ("combinable"[All Fields] OR "combinated"[All Fields] OR "combination"[All Fields] OR "combinational"[All Fields] OR "combinations"[All Fields] OR "combinative"[All Fields] OR "combine"[All Fields] OR "combined"[All Fields] OR "combines"[All Fields] OR "combining"[All Fields]) AND ("total disc replacement"[MeSH Terms] OR ("total"[All Fields] AND "disc"[All Fields] AND "replacement"[All Fields]) OR "total disc replacement"[All Fields] OR ("artificial"[All Fields] AND "disc"[All Fields] AND "replacement"[All Fields]) OR "artificial disc replacement"[All Fields]) AND (("radiograph"[All Fields] OR "radiographed"[All Fields] OR "radiographer"[All Fields] OR "radiographer s"[All Fields] OR "radiographers"[All Fields] OR "radiographic"[All Fields] OR "radiographical"[All Fields] OR "radiographically"[All Fields] OR "radiographics"[All Fields] OR "radiographing"[All Fields] OR "radiographs"[All Fields]) AND ("assess"[All Fields] OR "assessed"[All Fields] OR "assessement"[All Fields] OR "assesses"[All Fields] OR "assessing"[All Fields] OR "assessment"[All Fields] OR "assessment s"[All Fields] OR "assessments"[All Fields]) AND ("cervic"[All Fields] OR "cervicals"[All Fields] OR "cervices"[All Fields] OR "neck"[MeSH Terms] OR "neck"[All Fields] OR "cervical"[All Fields] OR "uterine cervicitis"[MeSH Terms] OR ("uterine"[All Fields] AND "cervicitis"[All Fields]) OR "uterine cervicitis"[All Fields] OR "cervicitis"[All Fields]) AND ("range of motion, articular"[MeSH Terms] OR ("range"[All Fields] AND "motion"[All Fields] AND "articular"[All Fields]) OR "articular range of motion"[All Fields] OR ("range"[All Fields] AND "motion"[All Fields]) OR "range of motion"[All Fields]))	3
**Comparator:** ACDF and TDR with Bone grafts, osteobiologics, bone matrix, DBM, autograft, allografts, cement, PMMA, TCP, BMP, BMA, stem cells, hydroxy apatite, ceramics, collagen, silicon, calcium sulphate, bioglass and composites	#19	("Bone Matrix"[Mesh] OR (Demineraliz* AND bone AND matri*))	5186

	#20	"bone transplantation"[MeSH Terms] OR ("bone"[All Fields] AND "transplantation"[All Fields]) OR "bone transplantation"[All Fields] OR ("bone"[All Fields] AND "graft"[All Fields]) OR "bone graft"[All Fields]	157,827
	#21	osteobiologics[All Fields]	42
	#22	("autografts"[MeSH Terms] OR "autografts"[All Fields])	8,481
	#23	(("ilium"[MeSH Terms] OR "ilium"[All Fields] OR ("iliac"[All Fields] AND "crest"[All Fields] AND "bone"[All Fields]) OR "iliac crest bone"[All Fields]) AND ("transplants"[MeSH Terms] OR "transplants"[All Fields] OR "graft"[All Fields]))	3,700
	#24	("allografts"[MeSH Terms] OR "allografts"[All Fields])	31,306
	#25	(("bone marrow"[MeSH Terms] OR ("bone"[All Fields] AND "marrow"[All Fields]) OR "bone marrow"[All Fields]) AND aspirate[All Fields]	9,305
	#26	"bone morphogenetic proteins"[MeSH Terms] OR ("bone"[All Fields] AND "morphogenetic"[All Fields] AND "proteins"[All Fields]) OR "bone morphogenetic proteins"[All Fields] OR ("bone"[All Fields] AND "morphogenetic"[All Fields] AND "protein"[All Fields]) OR "bone morphogenetic protein"[All Fields]	27,637
	#27	"platelet-rich plasma"[MeSH Terms] OR ("platelet-rich"[All Fields] AND "plasma"[All Fields]) OR "platelet-rich plasma"[All Fields] OR ("platelet"[All Fields] AND "rich"[All Fields] AND "plasma"[All Fields]) OR "platelet rich plasma"[All Fields]	12,224
	#28	("mesenchymal stem cells"[MeSH Terms] OR ("mesenchymal"[All Fields] AND "stem"[All Fields] AND "cells"[All Fields]) OR "mesenchymal stem cells"[All Fields])	65,715
	#29	("beta-tricalcium phosphate"[Supplementary Concept] OR "beta-tricalcium phosphate"[All Fields] OR "beta tcp"[All Fields])	3,469
	#30	(synthetic[All Fields] AND ("transplantation"[Subheading] OR "transplantation"[All Fields] OR "grafts"[All Fields] OR "transplants"[MeSH Terms] OR "transplants"[All Fields])) OR "cement"[All Fields] OR ("polymethyl methacrylate"[MeSH Terms] OR ("polymethyl"[All Fields] AND "methacrylate"[All Fields]) OR "polymethyl methacrylate"[All Fields] OR "pmma"[All Fields]) OR "tcp"[All Fields] OR (hydroxy[All Fields] AND ("apatites"[MeSH Terms] OR "apatites"[All Fields] OR "apatite"[All Fields])) OR ("ceramics"[MeSH Terms] OR "ceramics"[All Fields]) OR ("collagen"[MeSH Terms] OR "collagen"[All Fields]) OR ("silicon"[MeSH Terms] OR "silicon"[All Fields]) OR ("calcium sulphate"[All Fields] OR "calcium sulfate"[MeSH Terms] OR ("calcium"[All Fields] AND "sulfate"[All Fields]) OR "calcium sulfate"[All Fields]) OR ("Bioglass"[Supplementary Concept] OR "Bioglass"[All Fields] OR "bioglass"[All Fields]) OR "graft"[All Fields]) AND "composites"[All Fields])	6,085
	#31	#19 OR #20 OR #21 OR #22 OR #23 OR #24 OR #25 OR #26 OR #27 OR #28 OR #29 OR #30	6,571
	#27	#8 OR #9 OR #12 AND #31	0
**Outcomes:** Radiographical assessment of cervical range of motion, fusion rate, adjacent segment degeneration, post-operative complications, VAS pain, NDI, Odom criteria		("radiograph"[All Fields] OR "radiographed"[All Fields] OR "radiographer"[All Fields] OR "radiographer s"[All Fields] OR "radiographers"[All Fields] OR "radiographic"[All Fields] OR "radiographical"[All Fields] OR "radiographically"[All Fields] OR "radiographics"[All Fields] OR "radiographing"[All Fields] OR "radiographs"[All Fields]) AND ("assess"[All Fields] OR "assessed"[All Fields] OR "assessement"[All Fields] OR "assesses"[All Fields] OR "assessing"[All Fields] OR "assessment"[All Fields] OR "assessment s"[All Fields] OR "assessments"[All Fields]) AND ("cervic"[All Fields] OR "cervicals"[All Fields] OR "cervices"[All Fields] OR "neck"[MeSH Terms] OR "neck"[All Fields] OR "cervical"[All Fields] OR "uterine cervicitis"[MeSH Terms] OR ("uterine"[All Fields] AND "cervicitis"[All Fields]) OR "uterine cervicitis"[All Fields] OR "cervicitis"[All Fields]) AND ("range of motion, articular"[MeSH Terms] OR ("range"[All Fields] AND "motion"[All Fields] AND "articular"[All Fields]) OR "articular range of motion"[All Fields] OR ("range"[All Fields] AND "motion"[All Fields]) OR "range of motion"[All Fields])	503
		#8 OR #9 OR #12 AND radiographical assessment of cervical range	3

Appendix III

Table S1: Surgical construct

Non-comparative studies					
**Author**	**Levels operated**	**Surgical approach**	**Intervention Construct**	**Disc/Cage**	**Osteobiologic**
Barbagallo et al.[13]	2-4	Standard right-sided approach	HS: TDR & ACDF	ProDisc-C (Synthes Spine); Prestige LP, Bryan disc or carbon fiber reinforced polymer-CFRP or Cornerstone CFC cages	Demineralized bone matrix
Cardoso et al.[19]	2-3	Standard approach	HS: TDR & ACDF	Prestige ST, Mystique resorbable plating system with PEEK interbody spacer (Cornerstone), PEEK Prevail Cervical Interbody Device	BMP-2
Shi et al.[20]	3	Standard right-sided approach	HS: TDR & ACDF	1x ProDisc-C (Synthes Spine) & 2 cages (Stryker) or<br>2x ProDisc-C (Synthes Spine) & ZERO-P (Synthes Spine) or<br>2x ProDisc-C (Synthes Spine) & cage (Stryker)	NA

Comparative studies (HS versus ACDF or TDR)			I	C		I	C
Brotzki et al.[21]	NA	Standard approach	I1: HS (TDR)<br>I2: HS (DCI)	ACDF	I1: Cerkinetic TDR device<br>I2: DCI<br>C: Titanium mesh BENGAL cage	NA	NA
Jang et al.[22]	3	NA	HS: TDR & ACDF	ACDF	I: Active-C disc and Baguera C<br>C: Cervical plate system (Zephir)	NA	NA

Table S1: Continued

Author	Levels operated	Surgical approach	Intervention Construct		Disc/Cage	Osteobiologic	
**Comparative studies (HS versus ACDF or TDR)**			**I**	**C**		**I**	**C**
Xu et al.[23]	3	Smith-Robinson approach	I1: HS (2 discs, 1 cage)<br>I2: HS (1 disc, 2 cages)	C1: TDR<br>C2: ACDF	Prodisc-C (Depuy); poly-ether-etherketone cage MC+ (LDR Medical)	NA	NA
Ji et al.[24]	2	Smith-Robinson approach	HS: TDR & ACDF	ACDF	I: Mobi-C; Fidji cage<br>C: Cervical plate system (Zephir)	Bovine bone	Autogenous iliac-crest graft
Kang et al.[25]	3	Smith-Robinson approach	HS: TDR & ACDF	ACDF	I: ProDisc-C (Synthes Spine); cervical interbody fusion cage or ZERO-P (Synthes Spine)<br>C: Cervical plate system (Synthes Spine)	Iliac bone	Autogenous iliac-crest graft
Shin et al.[26]	2	Smith-Robinson approach	HS: TDR & ACDF	ACDF	I: Mobi-C disc; Fidji cage<br>C: Cervical plate system (Zephir)	Bovine bone	Iliac-crest graft
Wu et al.[27]	2-3	Standard right-sided approach	HS: TDR & ACDF	TDR	I: Zero-P implant<br>C: Prestige-LP	B-tricalcium phosphate or local bone graft	B-tricalcium phosphate or local bone graft
Xiong et al.[28]	2	Standard right-sided approach	HS: TDR & ACDF	ACDF	I: Mobi-C disc; ROI-C cage<br>C: 2x ROI-C cages	Allogeneic bones	Allogeneic bones

** NA: Not Available, I: intervention group, C: control group, ACDF: Anterior Cervical Discectomy and Fusion, TDR: Total disc replacement, HS: Hybrid surgery, DCI: dynamic cervical implant*

Appendix IV

Table S2: Radiological findings

Author	(Cervical) ROM (mean °)	Fusion rate (%)	Adjacent segment degeneration	Subsidence rate (%)	Postoperative complications
**Non-comparative studies**					
Barbagallo et al.[13]	Postoperative ROM at arthroplasty level: 8.3° (0°-15°)	NA	NA	NA	HO McAfee grade 2: N=1; Grade 4: N=1
Cardoso et al.[19]	Cervical ROM 2-level HS: preoperatively: 50.5° ± 11.9°, postoperatively: 39.5° ± 10.4°* Cervical ROM 3-level HS: preoperatively: 48.8° ± 12.7°, postoperatively: 36.0° ± 16.3°	100	None	NA	Dysphagia N=7, RLN palsy N=1
Shi et al.[20]	C2-C7 ROM preoperative 46.39° ± 2.41°, Last follow-up: 47.50° ± 4.59	Satisfactory at last follow-up	N=2 symptomatic	NA	HO: N=3 without need for further intervention Mild disc prosthesis migration (<3 mm): N=2
**Comparative studies (HS versus ACDF or TDR)**					
Brotzki et al.[21]	Overall decrease in ROM (NS)	ACDF Showed fusion	I1 produced the best results*	NA	HO: I1 best prognosis
Jang et al.[22]	Decreased compared to preoperative range in both groups* C2-C7 ROM differences 6 months after surgery*	I: 94.7* C: 70*	I: N=2 C: N=11	NA	I: Dysphagia N=1, plate migration N=1. I lower incidences of complications vs C* C: Dysphagia N=6, plate migration N=5, screw pull out N=2, screw breakage N=1, graft dislodgement N=2.

Table S2: Continued

Author	ROM (mean °)	Fusion rate (%)	Adjacent segment degeneration	Subsidence rate (%)	Postoperative complications
Xu et al.[23]	C2-C7 ROM decreased compared to preoperative in all groups* (NS)	NA	NS	NA	C2: N=1 second-stage surgery
Ji et al.[24]	C2–C7 ROM differed between groups at 2 and 3 years after surgery*	I: 88<br>C: 94	NA	NA	I: HO 39% (N=7)<br>C: HO 53% (N=9)
Kang et al.[25]	C2-C7 ROM I and C improvement relative to preoperative at follow-up: 1,3 and 6 months*<br>Difference between I and C during follow-up*	NA	I: None found at follow-up<br>C: N=1	C: N=1	I: HO N=1
Shin et al.[26]	I more rapid C2-C7 recovery than C*<br>I recovered to preoperative value, in contrast to C*	NA	NA	NA	None
Wu et al.[27]	C2-C7 ROM I vs C improvement relative to preoperative (NS)	I: 100<br>C: 100	NS	NA	I: Dysphagia N=1<br>C: Dysphagia N=1
Xiong et al.[28]	C2-C7 ROM I > C at last follow-up*	I: 100<br>C: 100	None	I: 27.08<br>C: 37.21	I: Dysphagia N=8,<br>HO Grade 3 N=2<br>C: Dysphagia N=9

I: intervention group, C: control group, ROM: range of motion, HO: heterotopic ossification, NA: not assessed in study, NS: No statistically significant difference between groups
**Statistically significant (p<0.05)*

Appendix V

Table S3: Clinical findings

Author	VAS	NDI (%)	Odom Criteria
**Non-comparative studies**			
Barbagallo et al.[13]	NA	Preoperative: 31.5, Postoperative: 13.3*	NA
Cardoso et al.[19]	NA	NA	NA
Shi et al.[20]	NA	Preoperative: 61.17<br>Last follow-up: 18.64*	NA
**Comparative studies (HS versus ACDF or TDR)**			
Brotzki et al.[21]	VAS arm I1 showed greatest reduction in VAS score*	Preoperative vs postoperative change biggest in I1* Improvements between preoperative and final follow-up among 3 groups (NS)	NA
Jang et al.[22]	Decrease in VAS pain arm in both groups* (NS)	Improvement in both groups* (NS)<br>I demonstrated better relief than C after surgery	Improvement in both groups* (NS)
Xu et al.[23]	NA	Larger improvement in I2*	NA
Ji et al.[24]	Postoperative neck pain was lower in I than in C at 1 and 3 years after surgery*	Improvement in both groups*<br>I exhibited better recovery than C after surgery*	NA
Kang et al.[25]	I and C improved scores relative to preoperative score* (NS)	I and C improved scores relative to preoperative score* (NS)	NA
Shin et al.[26]	Postoperative neck pain less I compared to C*	I better recovery than C*	NA
Wu et al.[27]	NS	NS	NA
Xiong et al.[28]	NS	NS	NA
